# Active ingredients of a person-centred intervention for people on HIV treatment: analysis of mixed methods trial data

**DOI:** 10.1186/s12879-017-2900-0

**Published:** 2018-01-10

**Authors:** Keira Lowther, Richard Harding, Victoria Simms, Aabid Ahmed, Zipporah Ali, Nancy Gikaara, Lorraine Sherr, Hellen Kariuki, Irene J. Higginson, Lucy Ellen Selman

**Affiliations:** 1 Department of Palliative Care, Policy and Rehabilitation, King’s College London, Cicely Saunders Institute, Bessemer Road, SdE59PJ, London, UK; 2 Dartington Service Design Lab, Lower Hood Barn, Dartington, Totnes TQ9 6AB UK; 3 London School of Hygiene and Tropical Medicine, Keppel Street, London, WC1E 7HT UK; 4 Bomu Hospital, Off Airport Road, P.O. BOX 95683, Mombasa, Soweto Kenya; 5 Kenya Hospices and Palliative Care Association, P.O Box 20854, Nairobi, 00202 Kenya; 6 Dept of Infection and Population Health, University College London, Rowland Hill Street, London, NW32PF UK; 7 Department of Medical Physiology, University of Nairobi, Chiromo Campus, PO BOX 30197 (00100), Nairobi, Kenya; 8 Bristol Randomised Controlled Trials Collaboration, School of Social and Community Medicine, University of Bristol, 39 Whatley Rd, Bristol, BS8 2PS UK

**Keywords:** HIV, Palliative care, Mixed methods, RCT, Psychosocial

## Abstract

**Background:**

A new model of care is required to meet the changing needs of people living with HIV (PLWH), particularly in low and middle-income countries, where prevalence is highest. We evaluated a palliative care intervention for PLWH in Mombasa, Kenya. Although we found no effect on pain (primary outcome), there was a positive effect on mental health (secondary outcome) in the intervention group. To inform replication and implementation, we have determined the active ingredients of the intervention and their mechanisms of action.

**Methods:**

We conducted a randomised controlled trial (RCT) with qualitative exit interviews in HIV clinic attenders. The intervention was delivered over 5 months, with a minimum of 7 clinical contacts. Longitudinal quantitative data on components of care received were analysed using area under the curve and logistic regression. Qualitative data were analysed using inductive and deductive thematic analysis.

**Results:**

Quantitative data analysis identified that intervention patients received more weak opioid, laxatives, discussion about spiritual worries, emotional support from staff for themselves and their families, time to talk about worries, discussion about future and planning ahead. Qualitative data analysis found that patients reported that having time to talk, appropriate pain medication and effective health education was of therapeutic value for their psychological well-being. Integration of mixed method findings suggest that positive effect in quantitative measures of mental health and well-being are attributable to the active ingredients of: appropriate medication, effective health education and counselling, and having time to talk in clinical encounters. Mechanisms of action include symptom relief, improved understanding of illness and treatment, and support focused on articulated concerns.

**Conclusions:**

Routine care must provide opportunities and means for existing clinical staff to make routine appointments more person-centred. This approach enabled staff to identify and manage multidimensional problems and provide tailored health education and counselling.

**Trial registration:**

ClinicalTrials.gov (NCT01608802). Registered 12th May 2012.

## Background

Due to treatment advances, HIV has been transformed from an acute infection to a chronic condition for those with access to antiretroviral therapy (ART) [1]. However, in resource-constrained settings where the disease burden is greatest, a paradigm shift is needed for health systems and providers to provide an adequate, sustainable response [2]. The current model of HIV care is delivered in overstretched systems with weak infrastructure and high numbers of patients, and these challenges have been associated with attrition from ART programmes [3]. Due to the efforts made to meet the UNAIDS 90–90–90 targets, these problems will be exacerbated by the projected increases in people living with HIV (PLWH) accessing ART [4].

HIV outpatients in low and middle income settings have a high burden of psychosocial and physical problems [5], evident from the point of diagnosis [6], and persisting alongside ART use [7]. These problems negatively affect patients’ ability to maintain ART adherence and their engagement with and retention in care [8], which potentially leads to viral resistance and its associated clinical and public health implications. A potentially appropriate model to address these challenges is palliative care. This holistic, person-centred approach has been shown to effectively reduce distress in people living with HIV, but the evidence primarily relates to the end of life, predates the development of highly active ART, and is from high income countries [9].

Palliative care for PLWH is a complex intervention, and therefore evaluation should not focus only on outcomes, but also on process data [10, 11]. Identifying the active ingredients of an intervention is key to understanding the causal mechanisms affecting observable outcomes, the extent to which evaluation findings can be extrapolated to other contexts, and which components of an intervention should be replicated to achieve its benefits [10, 11]. We aimed to identify the active ingredients and mechanisms of action of a nurse-led palliative care intervention for PLWH, in the context of a randomised controlled trial (RCT) of the intervention in Kenya [12, 13].

## Methods

### Study design

We conducted a parallel, two-arm, pragmatic open-label RCT with qualitative exit interviews [14]. The RCT found that, compared to standard care, a nurse-led palliative care intervention was of benefit in terms of the mental health dimension of quality of life, psychiatric morbidity and psychosocial problems, but not physical health [13]. During the trial, we conducted a mixed methods evaluation to determine how these effects were achieved, by identifying active ingredients of the intervention and their mechanisms of action. We report the results here. The methodology of the RCT, details of the intervention and trial findings are reported elsewhere [12–14]. The trial was registered with ClinicalTrials.gov (NCT01608802).

PLWH at an HIV clinic in Mombasa, Kenya, were screened according to the following eligibility criteria: adults on ART for at least 1 month, with a pain or symptom score of 3–5 (from a possible range of 0 (best) to 5 (worst)) on the African Palliative Care Association Palliative Outcome Scale (APOS) [15, 16]. Exclusion criteria were pain and symptoms caused entirely by an acute problem (duration <2 weeks), receiving ART for prevention of mother-to-child transmission of HIV, and not speaking Swahili or English. Following baseline data collection, participants were block-randomised to either intervention or control. The trial was un-blinded to participants and researchers, but blinded to the data analyst.

Qualitative exit interviews were conducted with 20 participants from the intervention group. A sample of 20 was judged as likely to achieve thematic saturation while allowing in-depth interrogation [17]. Sampling was in line with an explanatory sequential mixed methods design: we purposively selected participants to achieve a maximum variation sample based on individuals’ quantitative clinical response to the intervention. Clinical response was measured using a locally-validated, disease-specific measure of quality of life, the Mental Health Summary Score (MHSS) from the Medical Outcomes Study-HIV (MOS-HIV), in which a change of 10 points across a scale of 100 is considered clinically significant [18]. Participants were classed as “improving” if the study exit MHSS was more than 10 points higher than the baseline score, “deteriorating” if the MHSS at study exit was more than 10 points lower than the baseline score, and “static” otherwise.

### Data collection and outcome variable definition

Each measure was used to collect quantitative data at five monthly interviews throughout the study period of 4 months (0,1, 2, 3, 4 months). We used an adapted version of the 22-item version of the Client Services Receipt Inventory (CSRI) [19, 20] to measure the components of care patients received during the study period. This generated a binary outcome of whether a component of care had been received during the study period or not. We created a variable to indicate whether the client had ever received each component of care throughout the study. This variable was used in analysis. As described, we used the mental health sub scale of the MOS-HIV (MHSS) [18] to measure clinical response to the intervention. The MOS-HIV is a 35 item questionnaire that has been culturally adapted to African settings and HIV populations [21–23].

The same researcher who collected the quantitative data conducted qualitative interviews. Interviews were conducted between two to 6 months after exiting the trial, in a private location in the participant’s language of choice – Swahili or English – and digitally recorded. The topic guide explored physical, psychological, social and spiritual aspects of participants’ experiences of living with HIV before, during and after the intervention, and their experiences of the intervention. Participants were also shown a line graph of their psychological well-being throughout the study, measured at five time points at monthly intervals (T0-T4) using the MOS-HIV MHSS. This was used to prompt discussion about their experiences during the study. If they could remember, they were asked what was happening when their well-being visibly changed on the graph. Experienced translators transcribed the interviews verbatim and, where needed, translated them into English. The translations were quality-checked against the interview recordings by the bilingual interviewer.

### The intervention

Two experienced HIV clinic nurses employed by the ART clinic received 2 weeks’ full-time palliative care training. To enhance the person-centredness of care, the nurses used an assessment and care plan addressing patients’ physical, psychological, social and spiritual problems. Complex cases (e.g. refractory pain) were referred to specialist palliative care. An experienced local hospice nurse provided the nurses with weekly supervision and mentoring.

In addition to usual care, patients in the intervention arm met the trained nurse immediately following baseline interview and allocation, then at 2 weeks, 4 weeks and three subsequent monthly appointments.

### Standard care

Patients randomly allocated to the control arm continued to receive usual care from the HIV clinic, which consisted of monthly clinical appointments.

### Ethics

The study was approved by King’s College London Research Ethics Committee (BDM/10/11–31) and the Kenyan Medical Research Institute (KEMRI/RES/7/3/1). All participants gave written informed consent.

### Analysis

To determine and explore the active ingredients of the intervention and their mechanisms of action, quantitative and qualitative data were analysed separately subsequent to integration. Each was given equal importance during the analysis.

#### Quantitative data analysis

Analysis followed the intention to treat approach as set out in the analysis plan in the published trial protocol [14]. *P* values were two-tailed and an alpha level of 0.05 was used to assess statistical significance. Single missing items were imputed using horizontal mean method and last value carried forward [24]. All quantitative analyses were conducted using STATA version 10. The quantitative data were analysed to detect differences in components of care received by the intervention and control groups, and to identify associations between components of care and improvement in mental health in the intervention group. A Bonferroni correction for the 22 tests to be performed during this analysis indicated that a threshold for significance of 0.002 would be a conservative indication of a statistically significant association. However, because of concerns that use of Bonferroni corrections leads to increased type II errors, a less conservative threshold of *p* ≤ 0.01 was used as a threshold for statistical significance [25].

#### Differences between palliative care and standard care

Using the CSRI data, a graph displaying the components of care received in the palliative care and standard care groups was created for visual analysis. Next, chi square tests were performed to identify any significant differences in care components received by the two trial arms.

#### Components of the intervention associated with improvement

An individual-level summary statistic of longitudinal psychological well-being was created by estimating the area under the curve (AUC) of the MHSS of each participant over time. The variable had a non-parametric distribution which violated assumptions of many statistical tests such as simple or quantile regression. Therefore we recoded it into a binary variable, which we used as the outcome for univariate logistic regression analyses to test the association between psychological well-being over time and receipt of each component of care (the explanatory variables). We created this binary variable using the median for the whole group (*n* = 114). This median was not significant from a clinical point of view, but it was used to maximise the size of subgroups and minimise confidence intervals in logistic regression.

#### Qualitative data analysis

To identify and explore aspects of the intervention described by participants as therapeutic, interview transcripts were analysed in NVIVO 10 using thematic analysis [26]. After familiarisation with the data, themes were generated through a combination of deductive coding - based on our aim of identifying the active ingredients of the intervention and their mechanisms of action - and inductive coding to capture additional themes arising. A hierarchy of major themes and sub-themes was created based on salience and conceptual coherence. The preliminary coding frame created by KL was reviewed, discussed and refined by the project team to ensure conceptual integrity, prior to application of the final coding frame to all transcripts (KL). To explore barriers to effectiveness of the intervention, deviant case analysis focused on qualitative data from the participants who remained static (*n* = 5) and the one participant in the intervention arm who deteriorated. A narrative account of the findings was drafted (KL, LS, RH) and refined with input from the project team.

## Results

Sample characteristics for the quantitative component of the study are presented in Table 1. Of the 2070 patients screened, 16% were eligible for inclusion into the trial, of whom 44.3% agreed to participate [12].

**Table 1:** Characteristics of the TOPCare study sample

	Entire sample (*n* = 120)	Control (*n* = 60)	Intervention (*n* = 60)
Female n(%)	97 (81)	49 (82)	48 (80)
Mean age in years (sd, range)	39 (8·9, 22–64)	40·5 (9·2, 22–64)	38·3 (8·2, 23–60)
Has a partner n (% yes)	76 (63)	36 (60)	40 (66·7)
Median no. children (IQR)	2 (1–4)	2.5 (1–4)	2 (1–4)
Median no. financial dependents (IQR)	3 (2–5)	4 (3–5·5)	3 (2–5)
Education n			
Never attended	10	6	4
<4 yrs. of school	3	1	2
Primary	76	35	41
Secondary	27	17	10
Diploma	4	1	3
Median years since HIV diagnosis (IQR)	3·5 (1·3–5·2)	4·7 (2·4–5·7)	2·6 (0·9–4·4)
Median years on ART (IQR)	2·5 (0·8–4·2)	3·0 (1·6–5·0)	1·6 (0·4–3·5)
Median CD4 count (cells/mm^3^, IQR)	358 (223–506)	343 (209–558)	359 (247–490)
Response to the intervention: n (%)	(*n* = 116)	(n = 60)	(*n* = 54)
Clinically significant improvement on MOS-HIV			
MHSS	63 (55)	31 (52)	31 (57)
Static	51 (45)	29 (48)	22 (41)
Deteriorated	1 (0.8)	0 (0)	1 (1.9)

The sample was predominantly female (81%), with a mean age of 39 years, and a median of three financial dependents (Table 1).

### Quantitative findings

#### Differences between palliative care and standard care

**Fig. 1:**
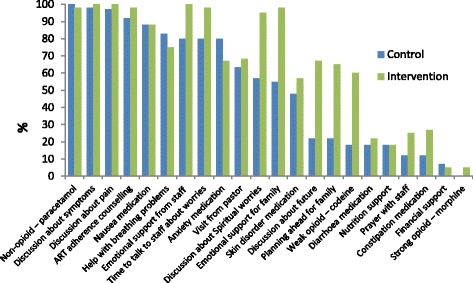
Comparison of components of care received by participants allocated to the intervention and control study arms

A visual comparison of the differences in the components of care received by participants in the two arms is depicted in Fig. 1.

Of the 22 care components in the CSRI, the proportion of participants in the intervention arm who received each component at least once during the study period was significantly higher for: discussion about spiritual worries (*X*^2^ = 24·1 *p* < 0·001); emotional support from staff (Fisher’s exact *p* < 0·001); time to talk to staff about worries (Fisher’s exact *p* < 0·001); emotional support for family (Fisher’s exact *p* < 0·001); weak opioids (*X*^2^ = 21.9 *p* < 0·001); discussion about the future (*X*^2^ = 24.6 *p* < 0·01); constipation medication (*X*^2^ = 4.36 *p* = 0·04); and support for the family in planning for the future (*X*^2^ = 22.9 *p* < 0·001) (Table 2).

**Table 2:** Numbers and percentage of participants receiving components of care at least once during 4 months of follow up by study arm

Service	Control % (n)	Intervention % (n)	*X*^2^ test and *p* value
Visit from spiritual leader	63·3 (38)	68·3 (41)	X^2^ = 0·33 *p* = 0·56
Discussion about spiritual worries	57 (34)	95 (57)	*X*^2^ = 24·1 *p* < 0·001
Prayer with staff	12 (7)	25 (15)	*X*^2^ = 3·56 *p* = 0·06
Emotional support from staff	80 (49)	100 (60)	Fisher’s exact *p* < 0·001
ART adherence counselling	92 (55)	98 (59)	Fisher’s exact *p* = 0·21
Time to talk to staff about worries	80 (48)	98 (59)	Fisher’s exact *p* < 0·001
Emotional support for family	55 (33)	98 (59)	Fisher’s exact *p* < 0·001
Discussion about pain	97 (58)	100 (60)	Fisher’s exact *p* = 0·50
Discussion about symptoms	98 (59)	100 (60)	Fisher’s exact *p* = 1·00
Non-opioid – paracetamol	100 (60)	98 (59)	Fisher’s exact *p* = 1·00
Weak opioid – codeine	18 (11)	60 (36)	*X*^2^ = 21·9 *p* < 0·001
Strong opioid – morphine	0 (0)	5 (3)	Fisher’s exact *p* = 0·24
Help with breathing problems	83 (50)	75 (45)	*X*^2^ = 1·26 *p* = 0·26
Anxiety medication	80 (48)	67 (40)	*X*^2^ = 2·72 *p* = 0·10
Nausea medication	88 (53)	88 (53)	*X*^2^ = 0·00 *p* = 1·00
Skin disorder medication	48 (29)	57 (34)	*X*^2^ = 0·84 *p* = 0·36
Diarrhoea medication	18 (11)	22 (13)	*X*^2^ = 0·21 *p* = 0·65
Constipation medication	12 (7)	27 (16)	*X*^2^ = 4·36 *p* = 0·04
Discussion about future	22 (13)	67 (40)	*X*^2^ = 24·6 *p* < 0·01
Planning ahead for family	22 (13)	65 (39)	*X*^2^ = 22·9 *p* < 0·001
Nutrition support	18 (30)	18 (30)	*X*^2^ = 0·00 *p* = 1·00
Financial support	7 (4)	5 (3)	Fisher’s exact *p* = 1·00

#### Components of the intervention associated with improvement

Almost all participants reported receiving emotional support from staff (100%), ART adherence counselling (98%), time to talk to staff about worries (98%), discussion with staff about pain (100%) and discussion about physical symptoms (100%). Due to the lack of variation, analysis of these variables was not possible.

The median AUC estimate was 210.2 (IQR 186.6–230.0) for the 114 participants who had more than 1 observation. It was recoded into a binary variable with scores below the median indicating low MHSS over time. Among intervention arm participants the median AUC estimate was 216.1 (IQR 147.2–261.3). All intervention participants who received discussions about their spiritual worries, non-opioid analgesia and anti-emetics reported levels of psychological well-being higher than the median score. All participants who received morphine reported levels of psychological well-being lower than the median score. There was insufficient variation to produce a valid odds ratio for these exposure variables. No other components of care were associated with psychological well-being over time.

### Qualitative findings

The qualitative sample was predominately female (17/20), with a median of four financial dependants and low levels of education (Table 3). All trial participants invited for qualitative interview agreed. The mean time between study exit and interview was 4.2 months.

**Table 3:** Characteristics of intervention arm qualitative sample (*n* = 20)

Variable	Qualitative sample (*n* = 20)
Female n (%)	17 (85)
Mean age in years (sd, range)	39·5 (6·75, 28–54)
Has a partner n (% yes)	12 (60)
Median number of children (IQR)	2 (2–3)
Median number of financial dependents (IQR)	3 (2–4)
Education attainment n (%)	
None	2 (10)
4 years or less	2 (10)
Primary education	10 (50)
Secondary education	6 (30)
Diploma	0
Median years since HIV diagnosis (IQR)	3.8 (1.2–5.0)
Median years on ART (IQR)	2.8 (1.1–4.6)
Median CD4 count (cells/mm^3^, IQR)	341 (203–513)
Change in MOS-HIV MHSS over time:	
Clinically significant improvement	14 (70)
Static	5 (25)
Deteriorated	1 (5)

#### Components of the intervention reported to be therapeutic

Three active ingredients were identified through analysis of the qualitative data: i) appropriate medication, ii) health education and counselling, and iii) having time to talk. Associated mechanisms of action were symptom relief and improved function; improved insight and understanding; and articulated concerns and focussed support respectively.

### Appropriate medication

Long-standing pain was resolved through access to appropriate medication – weak opioids. Relief of physical pain and distress contributed to an overall improvement in well-being, and a return to previous levels of physical and social function:

*Whenever you found me to be sick, you got me the required medication free of charge. That helped me and kept me going for a long time… I started to feel like I had resurrected. I felt like I’d come out of the grave. ID125, female, 30 years, intervention*

The benefit of appropriate medication was enhanced by receiving health education and counselling:

*I was distressed but I came here and was put under medication, got better and went on well with my work… I was helped with guidance, counselling on the importance of sticking to medication… and I got healed*. *ID 132, female, 45 years,*

#### Mechanism of action: Symptom relief and improved function

Improved physical health meant participants could increasingly care for themselves, earn a living and provide for their dependants. This alleviated stress and contributed towards their positive self-image:

*The pain eased because of the drugs that I took… I can now move around, I can go to work and I can do anything… That was the most important thing:…my legs got well again and I started working, I felt like a normal human being. ID 123, male, 44 years*

#### Health education and counselling

The health education and counselling provided comprised advice on how to live with an HIV diagnosis in a positive way, including guidance on how to reduce sexual transmission, manage disclosure to the community and family, and cope with discrimination:

*What benefited me most was the guidance on how to live positively, commitment to church and being prayerful. That I should always pray when faced with difficulty, eat well, and I will have a long life. ID 132, female, 45 years*

Together with the study nurses, the participants were able to identify and address substantial unmet health education needs:

*[My health] kept on improving because there were many things that I had not known about that the nurses taught me. ID 120, female, 36 years*

#### Mechanism of action: Improved insight and understanding

Despite a median of 2·42 years on ART, participants described gaps in their knowledge about treatment. Increased insight and understanding helped participants take their medications as prescribed and make healthy decisions:

*The advice that you gave me really supported me. I started thinking positively about my life and believed that I could have a long life. ID 110, female, 28 years*

Access to the information needed to adequately self-care was associated with the alleviation of depressive symptoms:

*I was in depressive moods, but later, owing to the constant guidance you gave to me and my realisation of the worthiness thereof, I started getting committed in my mind and adhering to your instructions. ID 106, female, 39 years*

#### Having time to talk

The third active ingredient was having time to talk. The clinic reported that an appointment in standard care took less than 8 min, compared with approximately 45 min in the intervention appointments; the qualitative data reflected this. In their meetings with the intervention nurses, participants had time dedicated to talking through their problems and concerns, and help articulating their needs:

*I am better because I was listened to; I was helped. I was given advice, and so I left with something. ID 108, female, 37 years*

The time the participants had with study nurses compared favourably with their experience of usual care: in the clinic, a higher patient: staff ratio unavoidably meant reduced contact time:

*Here, we have more time with [the nurses]; they will not see you in a hurry like the other place [standard care clinic], because there are other people waiting. Here you will be seen; you will explain your problem. ID 108, female, 37 years*

#### Mechanism of action: Articulated concerns and focussed support

The increased time to talk in the appointments with study nurses meant participants’ concerns were more clearly articulated and the nurses gained a greater understanding of their problems than in the standard care clinic. Advice and care planning could therefore focus on effectively addressing the root causes of their primary concerns:

*There is a difference, because here you have a lot of time to be with the doctor, to talk to him/her and to have them instruct you. But on the other side [in the standard care clinic], not that the nurses are unwilling, but you personally feel… “Ah there are others who are waiting to come in”. So you may be having issues that you’d want addressed but you feel, “Ah, I’ll raise when I come next time”. But here you feel free to ask anything, without any problem. ID 120, female, 36 years,*

Because they felt listened to, participants’ interactions with staff were reportedly more fruitful, and their emotional, social and educational needs were addressed in a targeted way, benefitting their physical and psychological well-being.

#### Deviant case analysis: Barriers to effectiveness

Intervention arm participants whose quality of life remained static or deteriorated reported concurrent intractable physical or social problems which prevented them from fulfilling their social roles and led to financial difficulties. This in turn led to stress, which was a barrier to positive psychological and well-being:

*It was bad because I could... my mind could only think on how to get a job, how to get my daily meal, and my life was just that way and I went on that way. ID 124, female, 37 years*

### Integration of findings

Participants in the intervention arm received more emotional support for themselves and their family, and had more time to talk about their worries (including spiritual concerns) and discuss the future. Their families also received more support with planning. Patients who received the intervention also received more weak opioids (codeine) and laxatives than those allocated to the control arm. Mental health improvement over the study period in the intervention group was significantly associated with receipt of spiritual discussions, paracetamol and anti-emetics, and negatively associated with receipt of morphine.

The qualitative and quantitative findings converge strongly, suggesting that appropriate and effective medication, effective health education and counselling, and increased time to talk in clinical encounters are associated with improvements in psychological well-being. Barriers to the effectiveness of the intervention were perceived intractable physical, social or psychological problems. The importance of holistic care is reflected in patient descriptions of benefit across physical, psychological and social domains.

## Discussion

This study is an important contribution to our understanding of interventions to improve mental health and long term coping amongst PLWH, and of the value of mixed method approaches in the evaluation of complex interventions. Our findings strongly suggest that increased time taken in the structured intervention clinical encounter enabled the participants to talk about their problems in a way they had previously been unable to do. In addition, improved health education meant that medications were better understood and regimens therefore better adhered to.

Whilst effective medication, health education and counseling, and having time to talk were identified as active ingredients of the intervention, it is possible that the effects of medication and health education and counseling were mediated by having time to talk. This hypothesis suggests that the intervention offered opportunities for patients to reflect and gain health information, increasing insight and understanding, as well as to describe their symptoms in more detail, enabling healthcare providers to prescribe more accurately and effectively and hence provide better symptom relief. Our data suggested that a structured holistic approach with adequate time to deliver these integrated components is needed to ensure they are effectively delivered in a person-centred and participatory way.

Time to talk is often seen as unfeasible in busy clinics where the number of clients is rising due to the increasing life expectancy of PLWH and earlier initiation of ART [27]. However, elsewhere, initial evaluation shows the use of peer mentors, expert clients or other trained health workers to alleviate common mental disorders holds promise [28, 29]. This task-shifting approach frees up nurse contact time, whilst maintaining gains in psychological well-being. On the other hand, initial time to talk may be a time saving process, even in a busy clinic, as the benefits might prevent more time-intensive needs in the future. It is interesting that participants receiving usual care in this study also reported improvements in mental health, and attributed this to their interactions with the researcher [30].

Our findings also highlight the benefits of conducting mixed-method explorations of process data alongside RCTs of complex interventions. A sole focus on the quantitative outcomes would not have identified the mechanisms through which the intervention was effective. The qualitative data also identified further therapeutic benefits attributed to the research process itself [31]. This exemplifies and supports the MRC guidance on process evaluation as an essential component of the evaluation of complex interventions [11, 32]. The findings presented here improve our understanding of which components of this intervention should be implemented in clinical practice and studied in future research.

A limitation of the study is that it is not possible to clarify from the data the extent to which therapeutic benefit is attributable to either the content or the duration of the appointments with the intervention nurses. However, the qualitative data suggest that they cannot be mutually exclusive; effective intervention requires a structured person-centred approach with adequate time for delivery. There is also potential for our findings to be affected by recall bias as participants were asked to describe events from previous months. The exact nature of the discussions that were described as therapeutic by the intervention group and which contributed to the active ingredient of time to talk is not currently known. While both control and intervention groups received discussions about pain and symptoms, only the intervention group described these discussions as therapeutically beneficial.

Our findings indicate directions for further research to improve HIV care in resource-constrained settings. Research should further develop and evaluate sustainable and feasible models of care for PLWH in these settings, testing the effects of implementing the active ingredients we have identified and their applicability in other contexts. The effects on retention and adherence to treatment of integrating palliative care into routine services should also be evaluated. Should the active ingredients we have found effective be implemented, outcomes should be compared when it is delivered by staff with higher and lower qualifications, to evaluate whether it is in fact time saving in the long term to task shift.

## Conclusions

Based on our findings, we recommend a person-centred approach to routine outpatient care for people on ART, including implementation of sufficient time to talk, effective health education and counselling, and access to appropriate medication for PLWH to manage pain and symptoms. Integrating time to talk into the clinical encounter is particularly important given its potential mediating role, and could be provided by non-medical staff, in line with recommendations for improving retention and adherence in HIV care [33]. This model may enable a more holistic approach to care in resource-constrained settings and relieve the psychological, physical and social burden experienced by PLWH.

## Abbreviations

APOS: APCA African POS
ART: Antiretroviral Therapy
AUC: Area Under the Curve
CSRI: Client Services Receipt Inventory
HIV: Human Immunodeficiency Virus
KEMRI: Kenyan Medical Research Institute
MHSS: Mental Health Summary Score
MOS-HIV: Medical Outcomes Scale HIV
MRC: Medical Research Council
PLWH: People living with HIV/AIDS
RCT: Randomised Controlled Trial
UNAIDS: United Nations Program for HIV/AIDS
